# Older adults report cancellation or avoidance of medical care during the COVID-19 pandemic: results from the Longitudinal Aging Study Amsterdam

**DOI:** 10.1007/s41999-021-00514-3

**Published:** 2021-05-28

**Authors:** Noah A. Schuster, Sascha de Breij, Laura A. Schaap, Natasja M. van Schoor, Mike J. L. Peters, Renate T. de Jongh, Martijn Huisman, Emiel O. Hoogendijk

**Affiliations:** 1 Department of Epidemiology and Data Science, Amsterdam Public Health Research Institute, Amsterdam UMC, Location VU University Medical Center, P.O. Box 7057, 1007MB Amsterdam, The Netherlands; 2 Department of Health Sciences, Faculty of Science, Amsterdam Public Health Research Institute, Vrije Universiteit Amsterdam, Amsterdam, The Netherlands; 3 Department of Internal Medicine, Section Geriatrics, Amsterdam UMC, Location VU University Medical Center, Amsterdam, The Netherlands; 4 Department of Internal Medicine, Section Endocrinology, Amsterdam UMC, Location VU University Medical Center, Amsterdam, The Netherlands

**Keywords:** Older adults, COVID-19, Healthcare use, Lockdown, Multimorbidity

## Abstract

**Purpose:**

Delay of routine medical care during the COVID-19 pandemic may have serious consequences for the health and functioning of older adults. The aim of this study was to investigate whether older adults reported cancellation or avoidance of medical care during the first months of the COVID-19 pandemic, and to explore associations with health and socio-demographic characteristics.

**Methods:**

Cross-sectional data of 880 older adults aged ≥ 62 years (mean age 73.4 years, 50.3% female) were used from the COVID-19 questionnaire of the Longitudinal Aging Study Amsterdam, a cohort study among community-dwelling older adults in the Netherlands. Cancellation and avoidance of care were assessed by self-report, and covered questions on cancellation of primary care (general practitioner), cancellation of hospital outpatient care, and postponed help-seeking. Respondent characteristics included age, sex, educational level, loneliness, depression, anxiety, frailty, multimorbidity and information on quarantine.

**Results:**

35% of the sample reported cancellations due to the COVID-19 situation, either initiated by the respondent (12%) or by healthcare professionals (29%). Postponed help-seeking was reported by 8% of the sample. Multimorbidity was associated with healthcare-initiated cancellations (primary care OR = 1.92, 95% CI = 1.09–3.50; hospital OR = 1.86, 95% CI = 1.28–2.74) and respondent-initiated hospital outpatient cancellations (OR = 2.02, 95% CI = 1.04–4.12). Depressive symptoms were associated with postponed help-seeking (OR = 1.15, 95% CI = 1.06–1.24).

**Conclusion:**

About one third of the study sample reported cancellation or avoidance of medical care during the first months of the pandemic, and this was more common among those with multiple chronic conditions. How this impacts outcomes in the long term should be investigated in future research.

## Key summary points

**Aim:**

To investigate the extent to which Dutch older adults reported cancellation or avoidance of medical care during the first months of the COVID-19 pandemic.

**Findings:**

One third of the study sample reported cancellation or avoidance of medical care during the first months of the pandemic, and this was more common among those with multiple chronic conditions.

**Message:**

Delay of routine care during the pandemic may impact morbidity and related adverse outcomes in the long term, which should be monitored in future research.

## Introduction

In the first months of 2020, Europe became the epicentre of the coronavirus disease 2019 (COVID-19) outbreak. The disease, caused by SARS-CoV-2, quickly spread across many countries in Europe. In the Netherlands, the first case of COVID-19 was confirmed on 27 February 2020 [1]. A strong increase in the number of new cases was observed, and the first COVID-19-related fatalities were reported in the following weeks. Mid-March, the Dutch government decided to implement a so-called “intelligent lockdown” to contain the outbreak [2]. This lockdown consisted of several social distancing measures, such as the strong advice to stay at home, as well as the closure of public places such as schools, restaurants and gyms. Most measures were eased from mid-May, but people were still advised to keep 1.5 m distance from other persons and to stay at home as much as possible.

In the Netherlands, just like in many other countries, regular healthcare has been majorly affected during the COVID-19 pandemic. During the first wave of the pandemic, outpatient specialist visits were limited to non-deferrable ones and appointments were cancelled, postponed or converted to telemedicine. Furthermore, many people may have avoided to visit their general practitioner (GP) or hospitals, as a means of social distancing or for fear of COVID-19 infection [3]. The effects of the interruption of routine care are still unknown, but it has been suggested that it may contribute to increased morbidity and mortality related to acute and chronic health conditions [4].

Delay or avoidance of medical care during the COVID-19 pandemic may especially affect older populations, where chronic conditions and multimorbidity are highly prevalent [5]. Moreover, older adults – and other vulnerable groups–were stimulated by the Dutch government to self-quarantine to limit the risk of infection. During the lockdown period, many diseases may not have been detected, have been without necessary control, or have been worsened due to social isolation and physical deconditioning. This may have major consequences for health and functioning of older adults and could result in worse short- and long-term outcomes.

So far, publications on cancellation or avoidance of care during the COVID-19 pandemic have mainly focused on specific medical fields [6–8] or on a higher aggregation level (e.g., overall referral rates during the pandemic compared to before the pandemic [9, 10]). Very few studies were undertaken in older populations. One study from Germany used healthcare registration data of older adults, and observed a decrease in healthcare use and diagnosis of diseases during the lockdown period, compared to the same period 1 year earlier [11]. Another study among older adults > 75 years in the UK revealed no major problems with access to healthcare services during the first months of the COVID-19 pandemic [12]. However, this study was done among a small sample in one geographic area in England. Studies from the perspective of older adults based on a nationally representative sample are lacking.

The Longitudinal Aging Study Amsterdam (LASA) is an ongoing cohort study among a representative sample of older adults in the Netherlands [13, 14], with follow-up measurements approximately every 3 years. Because of the exceptional situation of the COVID-19 pandemic, an additional questionnaire was sent to participants in June 2020, to measure the impact of the pandemic on the daily lives of older adults [15]. The LASA COVID-19 questionnaire provides unique data from the perspective of the older person, as it includes questions on respondent- and healthcare-initiated cancellations and avoidance of care. Moreover, these data can be linked to personal characteristics, to find out whether there are specific groups that report more often cancellations or avoidance of care than others.

Using data from the LASA COVID-19 questionnaire, the aim of the current study was to investigate the extent to which older adults (age range 62–102 years) report cancellations or avoidance of medical care during the first months of the COVID-19 pandemic, and to explore associations with health and socio-demographic characteristics.

### Methods

#### Study sample

Data were used from LASA [13, 14]. This is an ongoing cohort study on physical, emotional, cognitive and social functioning among older adults in the Netherlands, which started in 1992. The study was initially based on a representative sample of older adults aged 55–84 years, which are interviewed approximately every 3 years, including clinical measurements. In 2002 and 2012, refresher cohorts aged 55–64 years were added to the study. Details on the sampling, measurements and data collection of LASA have been published previously [13, 14]. Just before the outbreak of the COVID-19 pandemic, a LASA measurement wave was completed (2018–2019), and the next measurement wave was foreseen for 2021–2022. Therefore, it was decided to add a measurement in between, to assess functioning of older adults during the COVID-19 pandemic. This was a postal questionnaire that was sent to LASA participants in June, 2020. The questionnaire included measures to assess the impact of the COVID-19 situation, as well as a selection of measures from regular LASA measurement waves. The LASA study, including the COVID-19 questionnaire, was approved by the medical ethics committee of the VU University medical center. All participants provided written informed consent.

The COVID-19 questionnaire was sent by postal mail to eligible LASA participants on June 8, 2020, just after the first wave of the COVID-19 pandemic in the Netherlands. Participants were eligible if they participated in the last LASA measurement wave (2018–2019, *n* = 1701) and were still alive in March 2020 (*n* = 61 excluded). Moreover, participants were only selected if filling out the questionnaire was expected to be not too much of a burden (*n* = 151 excluded, because participants had short or proxy interviews in the 2018–2019 wave). Respondents could choose to fill out the questionnaire online (digital questionnaire). The oldest respondents (aged 80 +), who initially did not respond and for whom filling out a questionnaire was too difficult, were offered to answer the questions in a telephone interview. Of 1485 respondents who were approached, 1128 (76%) participated. Data were recorded between 9 June 2020 and 8 October 2020, of which 99% was received before the end of August 2020. The data were based on 909 written questionnaires, 198 digital questionnaires, and 21 telephone interviews. More details on the LASA COVID-19 questionnaire, including non-response analyses, can be found in a previous publication [15].

For the current study, we used data from the LASA COVID-19 questionnaire, as well as for some variables (multimorbidity, frailty) data from the 2018–2019 measurement wave, when information was not included in the LASA COVID-19 questionnaire. After excluding participants with missing data on the outcome measures (*n* = 90) or covariates (*n* = 158), the final analytical sample consisted of 880 participants.

#### Measures

In this study, cancellation and avoidance of medical care during the pandemic were measured by asking questions on the experience of healthcare-initiated cancellations of scheduled appointments, respondent-initiated cancellations of scheduled appointments, and postponed help-seeking. Respondents were asked whether a scheduled appointment had been cancelled by a healthcare professional because of the COVID-19 situation. This was asked separately for primary care (GP) and hospital outpatient care (medical specialist). Respondents were also asked if they cancelled a scheduled appointment themselves because of the COVID-19 situation. Again, this was assessed separately for primary care (GP) and hospital outpatient care. Postponed help-seeking was assessed by asking respondents whether they, because of the COVID-19 situation, did not seek for help in case of physical or mental health problems. All outcome variables were binary (no/yes).

Covariates included sex, age, education, functional limitations, loneliness, depressive symptoms, anxiety, multimorbidity, frailty, and information on quarantine. Continuous variables with a non-linear association with outcomes were categorised (age, loneliness, anxiety, and frailty). Three age groups were distinguished: < 70, 70–79 and ≥ 80 years. The highest level of completed education was categorised in three groups: low (elementary school or less), medium (lower vocational or general intermediate education), and high (intermediate vocational education, general secondary school, higher vocational education, college or university). Functional limitations were assessed by asking respondents about difficulties in performing seven basic activities of daily living: climbing the stairs, dressing and undressing, sitting down and getting up from a chair, cutting one`s own toenails, walking 5 minutes outdoors without resting, using transportation, and bathing. Mild functional limitations were defined as having difficulty with at least one activity, severe functional limitations were considered present if a respondent could not perform at least one activity or only with help. Loneliness was measured by the De Jong Gierveld Loneliness Scale (0–11). A cut-point of 3 or higher was applied to indicate the presence of loneliness [16]. Depressive symptoms were measured using the Center for Epidemiologic Studies Depression Scale (CES-D) [17], which was used as a continuous measure (0–60). Anxiety symptoms were examined by the Hospital Anxiety and Depression Scale (HADS-A, 0–21). A cut-off of ≥ 8 indicated the presence of anxiety [18]. Respondents were asked about the presence of chronic diseases: heart disease, arterial disease, diabetes mellitus, stroke, arthritis (rheumatoid arthritis or osteoarthritis), cancer, lung disease (asthma or chronic obstructive pulmonary disease), and a maximum of two other chronic diseases. Multimorbidity was defined as the presence of two or more diseases [19]. Frailty was operationalised using a 32-item frailty index based on the deficit accumulation approach, which has previously been developed and validated in LASA [20]. A commonly used cut-point of ≥ 0.25 was applied to indicate the presence of frailty [21]. Finally, respondents were asked whether they had been in quarantine (no/yes).

#### Statistical analysis

Descriptive analyses were performed to characterise the study sample. Proportions were reported for categorical variables and medians (IQRs) were reported for depressive symptoms because of its non-normal distribution. All characteristics were shown by cancellation or avoidance of care status. This was done for any healthcare-initiated cancellation of appointments, any respondent-initiated cancellation, postponed help-seeking by respondents, and for cancellations related to specific settings (primary care vs. hospital). Differences between groups were determined using Chi-square tests for categorical variables or Mann–Whitney tests for medians. Next, multivariable logistic regression analyses were performed to study the associations between respondent characteristics and healthcare-initiated cancellations, respondent-initiated cancellations, and postponed help-seeking. Cancellations were analysed separately for primary care and hospital outpatient appointments. All analyses were done in R version 4.0.2.

### Results

**Fig. 1:**
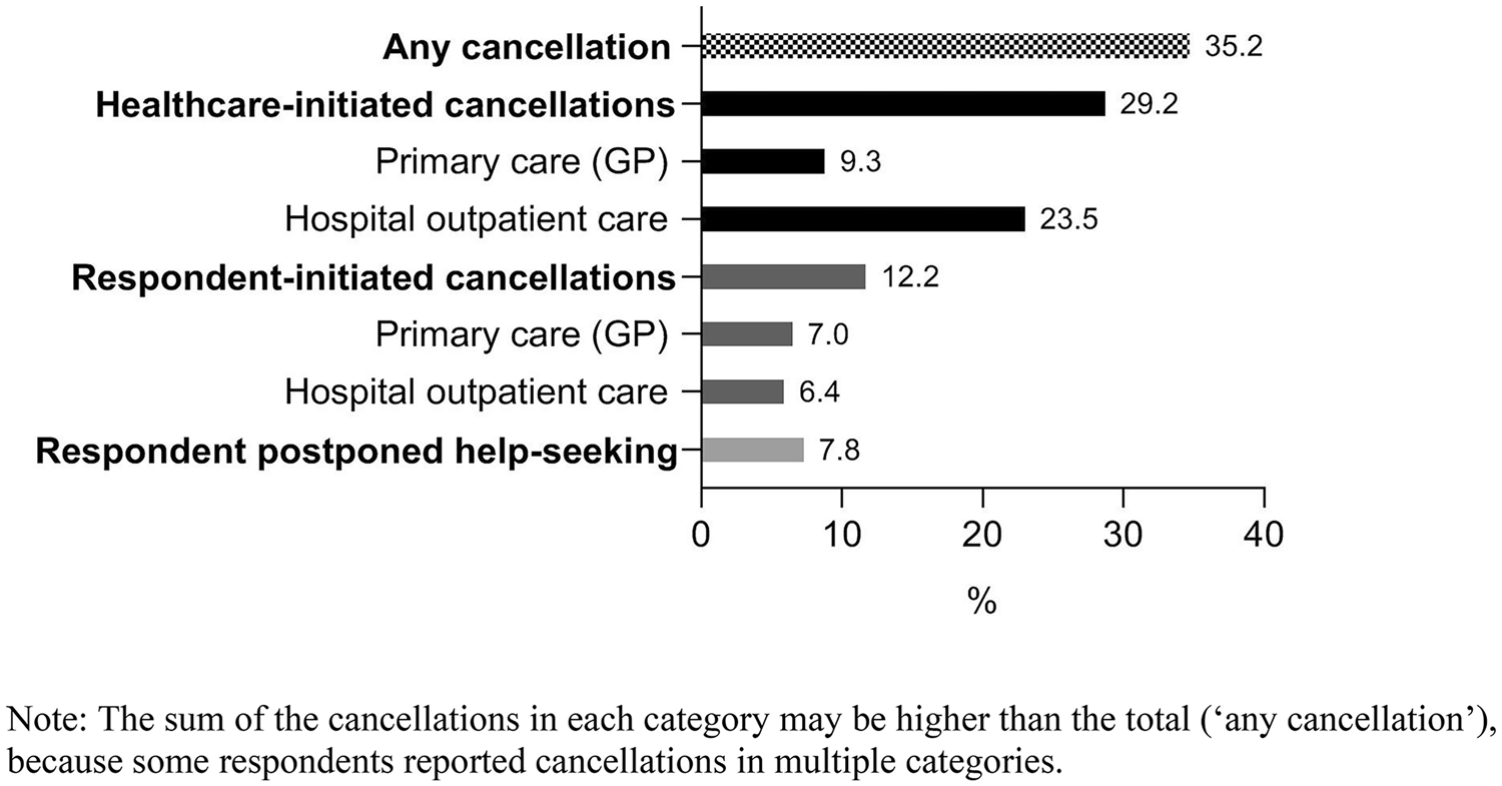
Cancellation or avoidance of medical care during the first months of the COVID-19 pandemic

Figure 1 and Table 1 show the characteristics of the study sample. The age range of the sample was 62–102 years (mean age = 73.4, SD = 7.2), 50.3% were female, and 57.8% were higher educated. Cancellations of care because of the COVID-19 situation were reported by 35.2% of the sample. Of the participants, 29.2% experienced a healthcare-initiated cancellation, 9.3% in primary care and 23.5% in hospital outpatient care. Furthermore, 12.2% of the sample reported to have cancelled an appointment themselves because of the COVID-19 situation, 7.0% cancelled a primary care appointment and 6.4% cancelled a hospital outpatient appointment. Postponement of help-seeking was reported by 7.8% of the sample.

**Table 1:** Study sample characteristics: Total sample and stratified by cancellation or avoidance of medical care during the first months of the COVID-19 pandemic

Characteristics	Total	Cancellation or avoidance of medical care								
		Healthcare-initiated cancellation			Respondent-initiated cancellation			Respondent postponed help-seeking		
		No	Yes	*p*	No	Yes	*p*	No	Yes	*p*
	*n* = 880	*n* = 623	*n* = 257		*n* = 773	*n* = 107		*n* = 811	*n* = 69	
Sex, % female	50.3	47.7	56.8	0.02	50.2	51.4	0.90	49.6	59.4	0.15
Age, 62–102 years										
< 70 years, %	36.4	35.5	38.5	0.68	36.0	39.3	0.66	36.4	36.2	0.66
70 – 79 years, %	44.3	45.1	42.4		44.9	40.2		44.6	40.6	
≥ 80 years, %	19.3	19.4	19.1		19.1	20.6		19.0	23.2	
Education										
Low, %	10.7	10.8	10.5	0.33	10.7	10.3	0.08	10.2	15.9	0.07
Medium, %	31.5	32.9	28.0		32.7	22.4		32.4	20.3	
High, %	57.8	56.3	61.5		56.5	67.3		57.3	63.8	
Functional limitations										
No limitations, %	55.5	58.7	47.5	< 0.01	56.3	49.5	0.26	57.1	36.2	< 0.01
Mild, %	29.7	26.8	36.6		28.7	36.4		28.7	40.6	
Severe, %	14.9	14.4	16.0		15.0	14.0		14.2	23.2	
Loneliness, %	48.6	47.4	51.8	0.27	48.9	46.7	0.75	47.7	59.4	0.08
Depressive symptoms, median (IQR)	5 (5)	5 (4)	6 (6)	< 0.01	5 (4)	6 (6.5)	< 0.01	5 (4)	8 (6)	< 0.01
Anxiety (HADS-A > 7), %	10.6	9.5	13.2	0.13	10.3	12.1	0.69	9.2	26.1	< 0.01
Multimorbidity, %	63.6	58.7	75.5	< 0.01	62.0	75.7	< 0.01	62.6	75.4	0.05
Frailty (FI ≥ 0.25), %	19.0	17.8	21.8	0.20	18.5	22.4	0.40	17.9	31.9	< 0.01
Quarantine, % yes	12.4	11.2	15.2	0.13	11.1	21.5	< 0.01	12.0	17.4	0.26

*p* values based on χ^2^ tests and Mann–Whitney tests

*IQR *inter quartile range

Table 1 also shows the characteristics of the study sample by cancellation or avoidance of care status. Participants with a healthcare-initiated cancellation were more often female, and had more functional limitations, more depressive symptoms and more multimorbidity, compared to older adults without cancellations. Respondents who cancelled an appointment themselves had more depressive symptoms, more multimorbidity, and had more often been in quarantine. Respondents who postponed help-seeking had more functional limitations, more depressive symptoms, and higher rates of anxiety, multimorbidity and frailty, compared to those who did not report postponed help-seeking. In Table 2, the characteristics were stratified by healthcare setting. For primary care, differences between those with and without cancellations were observed for functional limitations, multimorbidity, depressive symptoms, and quarantine status. For hospital care, cancellation differences were observed with regard to sex, functional limitations, depressive symptoms and multimorbidity.

**Table 2:** Cancellation or avoidance of medical care during the first months of the COVID-19 pandemic by healthcare setting

Characteristics	Primary care appointment						Hospital outpatient appointment					
	GP-initiated cancellation			Respondent-initiated cancellation			Hospital-initiated cancellation			Respondent-initiated cancellation		
	No	Yes	*p*	No	Yes	*p*	No	Yes	*p*	No	Yes	*p*
	*n* = 798	*n* = 82		*n* = 820	*n* = 60		*n* = 673	*n* = 207		*n* = 824	n = 56	
Sex, % female	50.5	48.8	0.86	50.0	55.0	0.54	47.3	60.4	< 0.01	50.7	44.6	0.46
Age, 62–102 years												
< 70 years, %	36.0	40.2	0.72	35.9	43.3	0.12	36.0	37.7	0.89	36.4	35.7	0.99
70 – 79 years, %	44.5	42.7		45.2	31.7		44.7	43.0		44.3	44.6	
≥ 80 years, %	19.5	17.1		18.9	25.0		19.3	19.3		19.3	19.6	
Education												
Low, %	10.4	13.4	0.52	10.4	15.0	0.27	10.4	11.6	0.16	10.8	8.9	0.09
Medium, %	31.2	34.1		32.1	23.3		33.1	26.1		32.3	19.6	
High, %	58.4	52.4		57.6	61.7		56.5	62.3		56.9	71.4	
Functional limitations												
No limitations, %	57.3	37.8	< 0.01	56.5	41.7	0.04	57.4	49.3	0.04	55.3	57.1	0.87
Mild, %	28.8	37.8		28.7	43.3		27.5	36.7		29.6	30.4	
Severe, %	13.9	24.4		14.9	15.0		15.2	14.0		15.0	12.5	
Loneliness, %	48.2	52.4	0.54	49.1	41.7	0.32	47.4	52.7	0.21	48.2	55.4	0.37
Depressive symptoms, median (IQR)	5 (5)	6 (5)	0.10	5 (4)	6 (6.25)	0.04	5 (4)	6 (6)	< 0.01	5 (4)	6 (6.25)	0.03
Anxiety ( HADS-A > 7), %	10.3	13.4	0.49	10.6	10.0	1.00	9.5	14.0	0.09	10.1	17.9	0.11
Multimorbidity, %	62.2	78.0	< 0.01	62.8	75.0	0.08	60.2	74.9	< 0.01	62.7	76.8	0.05
Frailty (FI ≥ 0.25), %	18.3	25.6	0.14	18.8	21.7	0.70	17.8	22.7	0.14	18.6	25.0	0.31
Quarantine, % yes	11.8	18.3	0.13	11.3	26.7	< 0.01	11.6	15.0	0.24	12.0	17.9	0.28

*p *values based on χ^2^ tests and Mann–Whitney tests

*IQR *inter quartile range

The results of the multivariable logistic regression analyses (Table 3) show which characteristics were associated with cancellation or avoidance of care during the COVID-19 pandemic, when accounting for all covariates. Age (OR ≥ 80 years = 0.41, 95% CI = 0.19–0.85), mild functional limitations (OR = 2.07, 95% CI = 1.17–3.67), severe functional limitations (OR = 3.49, 95% CI = 1.57–7.60), and multimorbidity (OR = 1.92, 95% CI = 1.09–3.50) were associated with healthcare-initiated cancellations in primary care. Being female (OR = 1.66, 95% CI = 1.19–2.32) and multimorbidity (OR = 1.86, 95% CI = 1.28–2.74) were indicators of healthcare-initiated cancellations in hospital outpatient care. Age (OR 70–79 years = 0.51, 95% CI = 0.26–0.95), mild functional limitations (OR = 1.90, 95% CI = 1.01–3.58), depressive symptoms (OR = 1.09, 95% CI = 1.00–1.19), and quarantine (OR = 2.47, 95% CI = 1.28–4.55) showed associations with respondent-initiated cancellations in primary care, whereas multimorbidity (OR = 2.02, 95% CI = 1.04–4.12) was associated with respondent-initiated cancellations in hospital outpatient care. Finally, depressive symptoms were a statistically significant indicator of postponed help-seeking (OR = 1.15, 95% CI = 1.06–1.24).

**Table 3:** Multivariable logistic regression analyses: associations between respondent characteristics and cancellation or avoidance of care

	Healthcare-initiated cancellation		Respondent-initiated cancellation		Postponed help-seeking
	Primary care appointment	Hospital outpatient appointment	Primary care appointment	Hospital outpatient appointment	
	OR (95% CI)	OR (95% CI)	OR (95% CI)	OR (95% CI)	OR (95% CI)
Sex (female)	0.822 (0.507–1.330)	1.657 (1.190–2.316)**	1.048 (0.602–1.835)	0.701 (0.393–1.238)	1.208 (0.711–2.070)
Age					
< 70 years (ref.)	1.00	1.00	1.00	1.00	1.00
70 – 79 years	0.633 (0.371–1.078)	0.821 (0.568–1.186)	0.506 (0.263–0.953)*	1.019 (0.545–1.928)	0.781 (0.429–1.427)
≥ 80 years	0.413 (0.192–0.847)*	0.817 (0.496–1.331)	0.864 (0.402–1.801)	1.111 (0.465–2.536)	0.768 (0.356–1.613)
Education					
Low (ref.)	1.00	1.00	1.00	1.00	1.00
Medium	1.066 (0.502–2.406)	0.757 (0.430–1.358)	0.493 (0.200–1.265)	0.833 (0.286–2.770)	0.510 (0.214–1.245)
High	0.823 (0.403–1.805)	1.141 (0.678–1.974)	0.740 (0.341–1.752)	1.614 (0.648–4.919)	0.936 (0.449–2.096)
Functional limitations					
No limitations (ref.)	1.00	1.00	1.00	1.00	1.00
Mild	2.072 (1.171–3.668)*	1.317 (0.896–1.930)	1.901 (1.009–3.583)*	0.676 (0.335–1.317)	1.825 (0.980–3.402)
Severe	3.487 (1.572–7.603)**	0.768 (0.410–1.407)	1.051 (0.353–2.903)	0.388 (0.123–1.097)	1.530 (0.616–3.685)
Loneliness	1.101 (0.665–1.822)	1.132 (0.801–1.598)	0.598 (0.327–1.073)	1.079 (0.594–1.966)	0.951 (0.542–1.672)
Depressive symptoms	0.990 (0.913–1.069)	1.027 (0.973–1.084)	1.093 (1.000–1.189)*	1.070 (0.978–1.165)	1.146 (1.060–1.237)***
Anxiety (HADS-A > 7)	1.185 (0.473–2.842)	1.019 (0.548–1.871)	0.486 (0.149–1.401)	1.041 (0.382–2.715)	1.066 (0.471–2.364)
Multimorbidity	1.918 (1.092–3.502)*	1.864 (1.282–2.737)**	1.694 (0.902–3.323)	2.018 (1.043–4.115)*	1.269 (0.685–2.428)
Frailty (FI ≥ 0.25)	0.773 (0.383–1.519)	1.025 (0.619–1.687)	0.774 (0.327–1.745)	1.490 (0.642–3.357)	0.931 (0.447–1.900)
Quarantine	1.609 (0.838–2.930)	1.145 (0.711–1.807)	2.465 (1.283–4.545)**	1.446 (0.656–2.923)	1.165 (0.564–2.239)

*OR *odds ratio, *CI *confidence interval, *ref. * reference category

* < 0.05

** < 0.01

*** < 0.001

### Discussion

Using data from LASA, a cohort study of community-dwelling older adults in the Netherlands, this study provides insight into cancellation and avoidance of medical care during the first months of the COVID-19 pandemic. The results indicated that about one third of the general older population reported cancellations of appointments in primary care or hospital outpatient care, which were more often initiated by healthcare professionals than by respondents. The analyses also revealed that some health characteristics were associated with cancellation or avoidance of care. People with multimorbidity experienced more often healthcare-initiated cancellations in primary care and hospital outpatient care, as well as respondent-initiated hospital outpatient cancellations. Furthermore, postponed help-seeking for physical and mental health problems was present in 8% of the sample, and was linked to a higher number of depressive symptoms.

Our results extend previous research that was done in Germany based on medical record data of older adults [11]. This previous study showed that GP consultations and referrals to hospital specialist outpatient care decreased substantially (up to -39%) during the lockdown in the first wave of the pandemic. Our study investigates the same topic from the perspective of the older person and adds further information by linking data on cancellation or avoidance of medical care with demographic and health characteristics of respondents. We found that multimorbidity was the factor that was most consistently associated with cancellation or avoidance of care. However, other associations are also noteworthy. For example, older adults who had been in quarantine more often cancelled a GP appointment themselves. Interestingly, a higher age was associated with less cancellations in primary care. This suggests that in primary care in the Netherlands, GPs have tried to maintain their care for the oldest old during the first wave of the pandemic.

The cancellation or avoidance of medical care as observed in our study may have serious implications for long-term outcomes in the older population. Especially the fact that older adults with multiple chronic diseases more often reported cancellations in primary care and hospital outpatient care could result in higher morbidity and related adverse health outcomes, such as acute events. So far, various studies have documented the detrimental effects of the COVID-19 pandemic on timely diagnosis and treatment of chronic conditions, such as osteoporosis, cardiovascular disease and cancer [6, 7, 22, 23]. However, the long-term impact of delay in care is still unknown and should be investigated in future research. Moreover, the impact may not be limited to physical health conditions. We observed an association between depressive symptoms and postponed help-seeking for physical and medical health complaints. Although recent research on changes in mental health during the first months of the COVID-19 pandemic did not observe changes in mental health among older adults in the Netherlands [24], postponed help-seeking could result in worse mental health outcomes in the long term. Lastly, it should be noted that not all cancellations or avoidance of care contribute to worse outcomes. For example, not all appointments with GPs are made because of severe health problems, and some health issues may improve over time without seeing a healthcare professional.

This was one of the first studies to investigate the impact of the COVID-19 pandemic on cancellation and avoidance of medical care from the perspective of the older person. Other strengths include the use of data from a large community-based sample, and the broad range of demographic and health characteristics that were studied in relation to the outcomes. Nevertheless, our study has some limitations. First, our measures are primarily based on self-report. Therefore, we were not able to compare the self-reported cancellations of appointments with more objective measures such as registration data from GP practices or hospitals. Second, we lack detailed information on the appointments that were cancelled, such as the reasons for consulting a healthcare professional. As a result, we do not know to what extent these include any acute care situations, for which the consequences of not seeking for help could be more severe. Third, some associations should be interpreted with caution, because of the cross-sectional design of the study and because of the fact that some groups have an a priori higher chance of cancellations. For example, older adults with multimorbidity have on average more appointments, increasing their risk of cancellation of appointments. Fourth, the current study made use of data from a follow-up measurement of an ongoing cohort study (LASA). Loss to follow-up in cohort studies may result in selection bias, as those who do not participate in follow-up measurements are often less healthy. Fifth, our results pertain to a sample of older adults in the Netherlands. Although we believe that our findings represent a general issue that was observed in many countries during the first wave of the COVID-19 pandemic, it remains to be seen to what extent our results are generalisable to countries with different healthcare systems. Finally, our study only covered the first wave of the pandemic. We do not know whether routine healthcare was affected in the same way in later waves of the pandemic. It is possible that over time, the impact on healthcare systems will become less strong, as systems will recover and adapt to the new situation. For example, face-to-face consultations may increasingly be replaced by teleconsultations, such as video visits. At the same time, a shift to telemedicine may not be feasible among older populations. For some groups, the access to telemedical services may be problematic due to a lack of experience with new technologies or because of disability [25].

In conclusion, using data from a large community-based sample in the Netherlands, this study showed that about one third of the older population reported cancellation or avoidance of medical care during the first months of the COVID-19 pandemic. Cancellations of appointments in primary care and hospital outpatient care were more common among those with multiple chronic conditions. How this impacts morbidity and related adverse outcomes in the long term should be investigated in future research.

## Data Availability

The datasets generated during the current study are not publicly available due to confidentiality, but the data underlying the results presented in this study are available from the Longitudinal Aging Study Amsterdam (LASA). Data of LASA, including data from the LASA COVID-19 questionnaire, may be requested for research purposes. More information on data requests can be found on the LASA website: www.lasa-vu.nl.
